# PHEVIR: an artificial intelligence algorithm that predicts the molecular role of pathogens in complex human diseases

**DOI:** 10.1038/s41598-022-25412-x

**Published:** 2022-12-03

**Authors:** Hongyi Zhou, Courtney Astore, Jeffrey Skolnick

**Affiliations:** Center for the Study of Systems Biology, School of Biological Sciences, Georgia Institute of Technology, 950 Atlantic Drive, N.W., Atlanta, GA 30332 USA

**Keywords:** Computational biology and bioinformatics, Infectious diseases

## Abstract

Infectious diseases are known to cause a wide variety of post-infection complications. However, it’s been challenging to identify which diseases are most associated with a given pathogen infection. Using the recently developed LeMeDISCO approach that predicts comorbid diseases associated with a given set of putative mode of action (MOA) proteins and pathogen-human protein interactomes, we developed PHEVIR, an algorithm which predicts the corresponding human disease comorbidities of 312 viruses and 57 bacteria. These predictions provide an understanding of the molecular bases of complications and means of identifying appropriate drug targets to treat them. As an illustration of its power, PHEVIR is applied to identify putative driver pathogens and corresponding human MOA proteins for Type 2 diabetes, atherosclerosis, Alzheimer’s disease, and inflammatory bowel disease. Additionally, we explore the origins of the oncogenicity/oncolyticity of certain pathogens and the relationship between heart disease and influenza. The full **PHEVIR** database is available at https://sites.gatech.edu/cssb/phevir/.

## Introduction

Infectious diseases have been a source of widespread, fatal outcomes throughout history^1^. As typified by COVID-19, pandemics in recent decades have become more frequent and deadly^1,2^. Yet, host–pathogen interactions are poorly characterized, and how they result in post-infection complications are not well-understood^3–7^. Host–pathogen interactome data provides an opportunity to assess putative diseases that can be at least partly attributed to a given set of host proteins that interact with a given pathogen. Characterizing such pathogen-disease associations can yield new areas of research and opportunities to develop targeted preventatives and therapeutics to not only treat the pathogenic infection itself but also prevent its potential downstream disease consequences^8^.

The COVID-19 pandemic has graphically illustrated numerous associations between SARS-CoV-2 and post-infection complications, such as loss of smell or unusual neurological symptoms^3,9^. Thus, it is obvious that infectious diseases can impact human health well beyond the initial virus infection. Although there is evidence that individuals with some common complex diseases are more susceptible to certain infectious diseases^10^, the contrary hasn’t been widely explored. That is, there has been limited research undergone to assess the association between viral or bacterial infections and the subsequent development of common complex diseases such as Type 2 diabetes, atherosclerosis, Alzheimer’s disease, and inflammatory bowel disease (IBD). For example, some cases of Alzheimer’s disease could be seeded by pathogen infection^11^. Furthermore, little is known about the post-infection complications associated with prevalent infectious diseases such as Influenza A and B, *E. coli*, Herpes simplex 1 and 2, salmonella, Epstein-Barr Virus (EBV) and clostridium difficile. For example, EBV infection increases the risk of developing autoimmune diseases such as IBD, Type 1 diabetes, and celiac disease^12^. More generally, perhaps pathogen infections play a greater role in causing complex human diseases than was previously appreciated.

Research has provided significant evidence that viruses and bacteria have oncogenic (cancer causing) and oncolytic (cancer treating) potential^13,14^. Indeed, eleven pathogens are now classified as carcinogenic according to the International Agency for Research on Cancer (IARC)^14,15^. Currently, approximately 12% of cancers have a known oncovirus association^14^. Both DNA and RNA viruses can contribute to cancer. For example, Epstein-Barr virus, a dsDNA virus, and human T-cell lymphotropic virus-1 (HTLV-1), an ssRNA-RT virus, are both implicated in some cancers. Some strains of human papillomavirus (HPV) cause cervical cancer^16^. There has also been speculation that SARS-CoV-2 might be an oncovirus^17^. Other pathogens could help in treating rather than causing cancers^13^. For examples, H5N1 influenza can induce cellular apoptosis^18^; measles viruses are oncolytic^19^, and herpes simplex virus 1(HSV-1) kills cancer cells^20^. However, despite such clear associations, it is unknown whether other pathogens also have a significant oncogenic/oncolytic potential. The problem is that oncogenic viruses might not give rise to cancers until a decade or longer following initial infection. As such, establishing the clinical connection between viral infection and the subsequent development of cancer is challenging.

To enhance the understanding of the mode of action (MOA) proteins driving the down-stream consequences associated with pathogen infection, we have developed the PHEVIR algorithm: disease comorbidities Predicted using Human–pathogEn interactomes for VIRulence. Here, we employ LeMeDISCO^21^, a recently developed tool that predicts on a proteomic scale human disease comorbidities, comorbidity enriched human MOA proteins and pathways given a pathogenic gene-human interactome set. At present, the pathogen-human interactome is provided by the HPIDB database^22^ but in practice any set of human–pathogen protein–protein interactions may be used. This work exploits the proteomic scale prediction of human disease MOA proteins for diseases identified by the artificial intelligence (AI) based method MEDICASCY^23^. The results of this analysis for 312 viruses and 57 bacteria are compiled in the PHEVIRdb whose goal is to guide and encourage research on human diseases that may be at least partly driven by pathogen infection. It is possible that it may take years post-infection for such complications to occur, or on the contrary, the predicted comorbidities explain how a preexisting disease might make one more susceptible to the particular comorbid infectious disease.

## Results

### Overview

The PHEVIR algorithm works as follows: Previously we employed the LeMeDISCO^21^ algorithm to predict disease co-morbidities based on a common set of mode of action (MOA) proteins. We assert that if a viral or bacterial protein interacts with these MOA proteins, it helps cause the corresponding comorbid diseases. The precision and recall rate of LeMeDISCO co-morbidity prediction on a large set of clinical observation data (~ 200,000 pairs of diseases) are 77.2 and 37.1%, respectively. On a variety of consensus datasets, in comparison to other molecular methods^24,25^, LeMeDISCO has an order of magnitude larger recall rate with similar precision^21^. For pathogen-cancer associated (either oncogenic or oncolytic) virus prediction, on a set of 13 viruses including 9 known oncogenic viruses, the recall rate is 66.7% with a precision of 100%^26^. We then examine the overall network of pathogen-diseases and focused our analysis on penetrant disease groups. Subsequently, pathogen-cancer and heart disease-flu relationships were examined in detail. For many of our predictions, we found literature evidence to support the predictions.

### Bacterial and viral induced human disease networks

A total of 39,393 significant pathogen-disease connections were identified (q-value < 0.05), of which 32,694 were virus—disease connections and 6699 were bacteria—disease connections. Of 3608 human diseases that might partially arise due to pathogen infections, 3285 unique diseases have at least one strong viral comorbidity. Similarly, 2405 unique diseases have at least one significant bacterial comorbidity. The top five viruses most connected to human diseases were Molluscum contagiosum virus subtype 1 that is comorbid to 1381 human diseases, Influenza A virus (strain A/Puerto Rico/8/1934 H1N1) that is comorbid to 1183 human diseases, Rubella virus (strain RA27/3 vaccine) that is comorbid to 1183 human diseases, Influenza A virus (strain A/Wilson-Smith/1933 H1N1) that is comorbid to 1137 human diseases, and Human immunodeficiency virus type 1 group M subtype B (isolate HXB2) that is comorbid to 1137 human diseases. The top five most connected bacteria in the network were *Helicobacter pylori* (strain ATCC 700392/26695) that is comorbid to 1080 human diseases, *Yersinia pestis* that is comorbid to 855 human diseases, *Staphylococcus aureus* that is comorbid to 578 human diseases, Streptococcus pyogenes serotype M1 that is comorbid to 428 human diseases, and *Mycoplasma pneumoniae* strain ATCC 29342/M129) that is comorbid to 406 human diseases. These results indicate that some pathogens are associated with up to one third of the diseases considered.

### Penetrant disease groups across pathogens

Tables 1 and 2 provide the numbers of comorbidities and their fractions in total pathogen-comorbidities for each ICD-10 main classification. The top three disease groups/classes with the greatest numbers of comorbid diseases to viruses were Neoplasms; Diseases of the eye and adnexa; and Diseases of the nervous system. The top three disease groups with the greatest numbers of comorbid diseases to bacteria were Certain infectious and parasitic diseases, Neoplasms and Diseases of the eye and adnexa. Apparently, the group of Certain infectious and parasitic diseases has the largest overall relative risk (RR, see Eq. 1 for definition) (1.22 with a *p* value of 6.46e−04 for viruses and 2.94 with a *p* value of 1.41e−113 for bacteria) that is consistent with its definition. The other frequent human diseases that are comorbid to both viruses and bacteria were Neoplasms. Both have a RR greater than 1 (RR = 1.54 with a *p* value of 1.90e−32 for viruses, and RR = 1.25 with a *p* value of 1.10e−06 for bacteria) compared to background causes of the diseases (see Tables 1 and 2). This association will be further addressed below. Another predicted high frequent comorbid disease group are Diseases of the eye and adnexa. However, it is not significantly risker than the background (RR = 1.001 with a *p* value of 0.98 for viruses and 1.06 with a *p* value of 0.33 for bacteria). Viral infections may cause irreversible neurological damage (RR = 1.12, *p* value = 0.06 for Diseases of the nervous system), possibly due to some of them being able to penetrate the blood brain barrier following an increased inflammatory response. This may lead to oxidative stress and dysregulation in producing sufficient energy^27,28^. It has also been speculated that viruses might contribute to or cause autoimmune diseases. Such viruses include Influenza A virus, Coxsackie B virus, rotavirus and herpes viruses^29^. Thus, assessing viral-induced autoimmunity is critical to preventing post-infection downstream complications.

**Table 1:** Comorbidities of 20 ICD-10 main classifications for viruses.

ICD-10_main_classification	Number of comorbidities	Fraction of comorbidities	Relative risk^a^	*p* value
Neoplasms	8089	0.247415	1.53645	1.90E-32
Diseases of the eye and adnexa	4952	0.151465	1.00089	0.983
Diseases of the nervous system	4581	0.140117	1.12094	0.062
Certain infectious and parasitic diseases	3756	0.114883	1.22271	6.46E-04
Endocrine, nutritional and metabolic diseases	3630	0.11103	0.938161	0.986
Diseases of the digestive system	1569	0.0479905	0.805347	0.989
Diseases of the blood and blood-forming organs and certain disorders involving the immune mechanism	1170	0.0357864	0.7873	0.981
Diseases of the circulatory system	921	0.0281703	0.434352	0.988
Congenital malformations, deformations and chromosomal abnormalities	879	0.0268857	0.194007	0.985
Diseases of the musculoskeletal system and connective tissue	671	0.0205236	0.540506	0.981
Diseases of the respiratory system	652	0.0199425	0.62028	0.986
Diseases of the genitourinary system	565	0.0172815	0.35427	0.979
Diseases of the skin and subcutaneous tissue	417	0.0127546	0.451164	0.984
Mental and behavioral disorders	183	0.00559736	0.166903	0.976
Symptoms, signs and abnormal clinical and laboratory findings, not elsewhere classified	175	0.00535266	0.772496	0.959
Diseases of the ear and mastoid process	128	0.00391509	0.0789142	0.981
Pregnancy, childbirth and the puerperium	121	0.00370098	0.667658	0.984
Factors influencing health status and contact with health services	91	0.00278339	0.91295	0.868
Certain conditions originating in the perinatal period	82	0.00250811	0.25855	0.977
Injury, poisoning and certain other consequences of external causes	62	0.00189637	0.570176	0.941

^a^See text for definition.

**Table 2:** Comorbidities of 20 ICD-10 main classifications for bacteria.

ICD-10_main_classification	Number of comorbidities	Fraction of comorbidities	Relative risk^a^	*p* value
Certain infectious and parasitic diseases	1849	0.276011	2.93761	1.41E-113
Neoplasms	1344	0.200627	1.24589	1.10E-06
Diseases of the eye and adnexa	1075	0.160472	1.06041	0.332
Endocrine, nutritional and metabolic diseases	558	0.083296	0.703822	0.976
Diseases of the digestive system	293	0.0437379	0.733983	0.963
Diseases of the genitourinary system	213	0.0317958	0.651814	0.969
Diseases of the circulatory system	198	0.0295567	0.455728	0.973
Diseases of the blood and blood-forming organs and certain disorders involving the immune mechanism	183	0.0273175	0.600985	0.964
Congenital malformations, deformations and chromosomal abnormalities	171	0.0255262	0.184197	0.971
Diseases of the musculoskeletal system and connective tissue	170	0.0253769	0.668321	0.968
Diseases of the nervous system	160	0.0238842	0.191073	0.973
Diseases of the skin and subcutaneous tissue	139	0.0207494	0.733958	0.972
Diseases of the respiratory system	131	0.0195552	0.608233	0.975
Mental and behavioural disorders	71	0.0105986	0.316031	0.972
Pregnancy, childbirth and the puerperium	46	0.0068667	1.23875	0.72
Symptoms, signs and abnormal clinical and laboratory findings, not elsewhere classified	33	0.00492611	0.710936	0.913
Factors influencing health status and contact with health services	23	0.00343335	1.12614	0.98
Diseases of the ear and mastoid process	22	0.00328407	0.0661952	0.988
Certain conditions originating in the perinatal period	15	0.00223914	0.230823	0.969
Injury, poisoning and certain other consequences of external causes	5	0.00074638	0.224412	0.885

^a^See text for definition.

### Common complex diseases and pathogens

Common complex diseases are diseases that are penetrant in the population and typically follow non-Mendelian patterns. They typically arise from a series of genetic and environmental factors that perhaps include infectious diseases. Four common complex diseases, Type 2 diabetes (T2D), Atherosclerosis, Inflammatory bowel disease (IBD) and Alzheimer’s diseases and their associations to infectious diseases were assessed. Table 3 demonstrates the top 10 pathogens for each disease. The complete lists including the MOA human proteins of all pathogens for these four diseases can be found in Supplementary Materials, Table S1.1–S1.4.

**Table 3:** Top 10 pathogens associated with 4 common complex diseases: Type 2 diabetes, Atherosclerosis, Inflammatory bowel disease, and Alzheimer’s disease.

Taxid	Pathogen name	Pathogen type	J-score	*p* value	q-value
**Type 2 diabetes**					
11678	Human immunodeficiency virus type 1 group M subtype B (isolate BH10)	Infections caused by ssRNA-RT viruses	0.0226	8.11E-06	0.00292
10360	Human cytomegalovirus (strain AD169)	Infections caused by dsDNA viruses	0.0169	0.000625	0.01437
11108	Hepatitis C virus genotype 1a (isolate H)	Infections caused by + ssRNA viruses	0.0166	0.000925	0.00867
333761	Human papillomavirus type 18	Infections caused by dsDNA viruses	0.0148	0.000578	0.0264
10600	Human papillomavirus type 6b	Infections caused by dsDNA viruses	0.0136	0.00495	0.0242
82830	Epstein-Barr virus (strain AG876)	Infections caused by dsDNA viruses	0.0134	0.00105	0.0145
**Atherosclerosis**					
333284	Hepatitis C virus genotype 1b (isolate Con1)	Infections caused by + ssRNA viruses	0.0179	0.00031	0.0445
10600	Human papillomavirus type 6b	Infections caused by dsDNA viruses	0.0174	0.00060	0.0183
41856	Hepatitis C virus genotype 1	Infections caused by + ssRNA viruses	0.0164	0.00144	0.0357
11103	Hepacivirus C	Infections caused by + ssRNA viruses	0.0145	1.17E-05	0.0004
85962	Helicobacter pylori (strain ATCC 700392/26695)	Infections caused by epsilon proteobacteria	0.0112	4.17E-05	0.0002
10280	Molluscum contagiosum virus subtype 1	Infections caused by dsDNA viruses	0.0070	0.0188	0.0492
**Inflammatory bowel disease**					
11706	Human immunodeficiency virus type 1 group M subtype B (isolate HXB2)	Infections caused by ssRNA-RT viruses	0.0274	4.94E-06	2.33E-05
211044	Influenza A virus (strain A/Puerto Rico/8/1934 H1N1)	Infections caused by -ssRNA viruses	0.0255	8.93E-05	0.0005
11044	Rubella virus (strain RA27/3 vaccine)	Infections caused by + ssRNA viruses	0.0255	9.06E-05	0.0005
632	Yersinia pestis	Infections caused by enterobacteria	0.0248	0.00015	0.0017
158611	Influenza A virus (A/Paris/908/97(H3N2))	Infections caused by -ssRNA viruses	0.0169	0.00327	0.0239
10280	Molluscum contagiosum virus subtype 1	Infections caused by dsDNA viruses	0.0157	2.43E-10	9.00E-10
382835	Influenza A virus (A/WSN/1933(H1N1))	Infections caused by -ssRNA viruses	0.0151	6.67E-05	0.0020
928302	Hepatitis B virus genotype C subtype ayr (isolate Human/Japan/Okamoto/-)	Infections caused by dsDNA-RT viruses	0.0131	0.00125	0.0107
11103	Hepacivirus C	Infections caused by + ssRNA viruses	0.0070	0.00022	0.0045
290579	Human immunodeficiency virus type 1 group M subtype B (isolate Lai)	Infections caused by ssRNA-RT viruses	0.0070	0.00036	0.0052
**Alzheimer’s disease**					
1891767	Simian virus 40	Infections caused by ssRNA-RT viruses	0.0185	0.00066	0.0053
211044	Influenza A virus (strain A/Puerto Rico/8/1934 H1N1)	Infections caused by -ssRNA viruses	0.0157	0.0044	0.0149
11044	Rubella virus (strain RA27/3 vaccine)	Infections caused by + ssRNA viruses	0.0157	0.0045	0.0151
381518	Influenza A virus (strain A/Wilson-Smith/1933 H1N1)	Infections caused by -ssRNA viruses	0.0156	0.0021	0.0074
796210	Bunyavirus La Crosse (isolate Human/United States/L78/1978)	Infections caused by -ssRNA viruses	0.0135	0.0032	0.0329
69156	Murid herpesvirus 1 (strain K181)	Infections caused by dsDNA viruses	0.0109	0.0018	0.0253
10915	Rotavirus A (strain RVA/Pig/United States/OSU/1977/G5P9[7])	Infections caused by dsRNA viruses	0.0091	7.81E-05	0.0013
31684	Simian immunodeficiency virus agm.grivet (isolate AGM gr-1)	Infections caused by ssRNA-RT viruses	0.0086	0.0028	0.0242
73475	Chicken anemia virus (isolate Germany Cuxhaven-1)	Infections caused by ssDNA viruses	0.0061	0.0019	0.0205
11707	Human immunodeficiency virus type 1 group M subtype B (isolate HXB3)	Infections caused by ssRNA-RT viruses	0.0060	0.0026	0.0153

Insulin resistance, a major characteristic of Type 2 diabetes, may be the consequence of frequent bouts of pathogen exposure and mild inflammatory response^30^. 6 significant viruses were predicted to be associated with Type 2 diabetes (q-value < 0.05) (see Table S1.1). Human immunodeficiency virus type 1 (HIV) is predicted to be the most significant virus associated with T2D. HIV-infected adults have a 3.8% higher incidence of diabetes mellitus than the general adult population^31^. Of the viruses associated with Type 2 diabetes, Epstein-Barr virus (strain AG876) (EBV) is prominent. The literature supports a link between Type 1 diabetes and EBV^32^. To identify the possible MOA proteins of EBV’s association with Type 2 diabetes, we examine the shared proteins of the EBV interactome with the MOA proteins of Type 2 diabetes predicted by MEDICASCY^23^. A total of 11 proteins are shared between them. MGST1 is associated with tissue damage that are part of diabetes^33^. All 6 viruses interact with CTSB. CTSB was found to contribute to Autophagy-related 7 (Atg7)-induced inflammatory response resulting in aggravation of lipotoxicity^34^ and increased T2D risk^35^.

Atherosclerosis is characterized by the formation of cholesterol plaque(s) in the walls of the arteries. 5 significant viruses and 1 significant bacteria are predicted to be associated with Atherosclerosis (see Table S1.2). The most significant is *helicobacter pylori*. Interestingly, it is significantly associated with subclinical coronary atherosclerosis in healthy subjects^36^. Human papillomavirus (HPV) is associated with increased prevalence of cardiovascular disease post-infection. This may be due to HPV increasing pro-inflammatory activity and altered lipid metabolism^37^. Three hepatitis C virus (HCV) strains were predicted to be associated with Atherosclerosis; HCV infection is known to be a risk factor for Atherosclerosis^38^. 4 of the 6 pathogens interact with human protein ITGB1. A bioinformatics study suggests that ITGB1 is a key gene associated with carotid atherosclerosis^39^.

Inflammatory Bowel Disease is an umbrella condition represented by Crohn’s disease and ulcerative colitis primarily characterized by intestinal inflammation. There are 10 significant viruses and 3 significant bacteria predicted to be associated with IBD (see Table S1.3). Literature suggests that dysregulation of intestinal mucosa may contribute to the pathogenesis of IBD^40^. Furthermore, gut microbiota play a major role in the pathogenesis of IBD as it may promote inflammation^41^. Some infectious diseases can alter the homeostasis of the gut microbiota, thus, contributing to the intestinal inflammation^42^. Influenza A virus (H1N1) is predicted to be significantly associated with IBD, a prediction supported by literature evidence^43^. Additionally, HIV is predicted to be significantly associated with IBD; indeed, HIV infection causes onset of Crohn’s disease^44^. Among the 90 unique MOA proteins of pathogens’ association with IBD (see Table S1.3, union of all MOA proteins), ITGB1 and GSN have the largest numbers (6 and 5 of 13) of interacting pathogens. ITGB1 plays an important role in the pathogenesis of IBD^45^; GSN is a potential biomarker for ulcerative colitis^46^.

Alzheimer’s disease (AD), a neurodegenerative disease characterized by memory loss and cognitive impairment, may result from the amyloid cascade or the tau hyperphosphorylation^47^. There is a theory that infections can seed some cases of AD^11^. Indeed, there are 10 significant viruses predicted to be associated with Alzheimer’s disease (see Table S1.4). Two strains of H1N1 are predicted to be significantly associated with AD. It has been shown by three studies that at least one flu vaccination is associated with a 17% *decrease* in AD incidence^48^. Among the 43 proteins interacting with H1N1, PPIA plays role in tau oligomerization and amyloid processing in AD^49^; RBBP7 is a mediator against neuronal loss in AD^50^. Simian virus 40 is predicted to be associated to AD. One study found that its antigen expression induces AD like pathology in mice^51^. Simian virus interacts with FBXW11 that is found to be related to AD alleviation^52^.

It is possible that infectious diseases may encourage damaging molecular processes in the specific human body/tissue yielding key characteristics of some common complex diseases such as persistent inflammation. It may also be that individuals have to encounter a series of these pathogens before subsequently developing a common complex disease.

### Prevalent pathogens and diseases

Next, we will explore the disease comorbidities associated with the following prevalent infectious pathogens: EBV (strain B95-8) (taxid 10377), Influenza A virus (strain A/New York/1682/2009(H1N1)) (taxid 643960), *Escherichia coli* (taxid 562) and Salmonella typhimurium (taxid 90371). Table 4 shows the top 10 comorbidities for each pathogen. Full lists including the MOA proteins are found in Supplementary Table S2.1–S2.4.

**Table 4:** Top 10 disease comorbidities associated with 4 common infectious pathogens: Epstein-Barr virus (EBV) (strain B95-8), Influenza A virus (strain A/New York/1682/2009(H1N1)), Escherichia coli (Taxid 562), and Salmonella typhimurium (Taxid 90371).

Disease	ICD-10 group	J-score	*p* value	q-value
**Epstein-Barr virus (strain B95-8) (taxid:10377)**				
Oligospermia	Diseases of the genitourinary system	0.060	0	0
Carbohydrate metabolic disorder	Endocrine, nutritional and metabolic diseases	0.056	1.79E−05	0.00034
Pellagra	Endocrine, nutritional and metabolic diseases	0.055	3.82E−05	0.00069
Pyridoxine deficiency anemia	Endocrine, nutritional and metabolic diseases	0.055	0.00012	0.00186
Gestational diabetes	Pregnancy, childbirth and the puerperium	0.055	1.49E−05	0.00029
Kwashiorkor	Endocrine, nutritional and metabolic diseases	0.054	0.00025	0.00363
Lipid metabolism disorder	Endocrine, nutritional and metabolic diseases	0.054	7.34E−05	0.00125
Beriberi	Endocrine, nutritional and metabolic diseases	0.054	0.00031	0.00434
Ariboflavinosis	Endocrine, nutritional and metabolic diseases	0.054	0.00031	0.00434
Osteomalacia	Diseases of the musculoskeletal system and connective tissue	0.054	0.00034	0.00476
**Influenza A virus (strain A/New York/1682/2009(H1N1)) (taxid:643960)**				
Tay-Sachs disease	Endocrine, nutritional and metabolic diseases	0.033	0	0
Sandhoff disease	Endocrine, nutritional and metabolic diseases	0.033	0	0
GM1 gangliosidosis	Endocrine, nutritional and metabolic diseases	0.033	0	0
Wolman disease	Endocrine, nutritional and metabolic diseases	0.033	0	0
Metachromatic leukodystrophy	Endocrine, nutritional and metabolic diseases	0.032	0	0
Hemoglobinopathy	Diseases of the blood and blood-forming organs and certain disorders involving the immune mechanism	0.032	0	0
Mucopolysaccharidosis III	Endocrine, nutritional and metabolic diseases	0.032	0	0
Coronary stenosis	Injury, poisoning and certain other consequences of external causes	0.032	0	0
Lysosomal storage disease	Endocrine, nutritional and metabolic diseases	0.031	0	0
Krabbe disease	Endocrine, nutritional and metabolic diseases	0.031	0	0
**Escherichia coli (taxid: 562)**				
Uveitis	Diseases of the eye and adnexa	0.0077	5.13E−08	3.70E−05
Pulmonary embolism	Diseases of the circulatory system	0.0077	5.63E−06	0.00073
Endocrine system disease	Endocrine, nutritional and metabolic diseases	0.0077	8.15E−09	1.47E−05
Nephrocalcinosis	Diseases of the genitourinary system	0.0074	3.76E−08	3.39E−05
Hypertension	Diseases of the circulatory system	0.0074	2.20E−09	7.94E−06
Iritis	Diseases of the eye and adnexa	0.0071	2.77E−08	3.33E−05
Gingival recession	Diseases of the digestive system	0.0068	8.31E−07	0.00037
Focal segmental glomerulosclerosis	Diseases of the genitourinary system	0.0067	4.59E−07	0.00024
Dental caries	Diseases of the digestive system	0.0066	1.18E−06	0.00042
Anovulation	Diseases of the genitourinary system	0.0065	1.54E−06	0.00047
**Salmonella typhimurium (taxid: 90371)**				
Pustulosis of palm and sole	Diseases of the skin and subcutaneous tissue	0.0039	1.79E−05	0.0073
Obstructive sleep apnea	Diseases of the nervous system	0.0038	2.20E−05	0.0073
psoriasis	Diseases of the skin and subcutaneous tissue	0.0038	2.29E−05	0.0073
Hyperglycemia	Symptoms, signs and abnormal clinical and laboratory findings, not elsewhere classified	0.0033	4.57E−05	0.0073
Chronic kidney disease	Diseases of the genitourinary system	0.0030	7.35E−06	0.0073
Uveitis	Diseases of the eye and adnexa	0.0026	1.77E−05	0.0073
Anterior uveitis	Diseases of the eye and adnexa	0.0026	5.43E−09	1.96E−05
Narcolepsy	Diseases of the nervous system	0.0025	2.32E−05	0.0073
Gingival recession	Diseases of the digestive system	0.0024	2.66E−05	0.0073
Dental caries	Diseases of the digestive system	0.0024	3.06E−05	0.0073

There were 408 significant comorbidities associated with EBV (strain B95-8) (see Table S2.1). Of these, 167 and 130 involve Endocrine nutritional and metabolic diseases and Neoplasms, respectively. Oligospermia, characterized by a low sperm count is the top associated comorbidity with EBV. Viral infections may contribute to male infertility via initiating inflammatory reactions that yield an increase in reactive oxygen species causing testicular damage^53^. Lipid metabolism disorder and carbohydrate metabolic disorder were predicted to be significant diseases associated with EBV. As another example, we examined the EBV interactome proteins of association with Gestational diabetes. We found 266 proteins that interact with EBV. The top 5 proteins prioritized by LeMeDISCO are STX7, STX10, CDIPT, GLIPR2, SNRPA. STX7 is upregulated in T2D^54^, and STX10 is differentially methylated in the offspring of women with maternal diabetes^55^. CDIPT was hypomethylated and up-regulated in the fetus of mice with Maternal Gestational Diabetes^56^. SNRPA was found to be associated with metabolic syndromes^57^.

There were 211 significant comorbidities associated with Influenza A virus (strain A/New York/1682/2009(H1N1) (see Table S2.2). The top 2 groups of diseases involve 33 Endocrine, nutritional and metabolic diseases and 26 Diseases of the blood and blood-forming organs and certain disorders involving the immune mechanism. The top 2 significant human comorbid diseases for Influenza A virus (flu) were Tay-Sachs disease (mutations in HEXA) and Sandhoff disease (mutations in HEXB) that are very similar rare genetic diseases. There is no direct interaction of flu with those two proteins. However, the human proteins in the flu-human interactome of HK1 protein interacts with both HEXA and HEXB^58^, and they share a pathway involving carbohydrate metabolism^59^. Another significant comorbid disease is coronary stenosis. Studies have suggested an association between influenza and cardiovascular diseases due to the activation of inflammatory pathways^7^. We shall examine this in more detail in the following “Heart disease and flu” section. There were several other rare diseases predicted to be associated with Influenza A. Some involve the immune mechanism and result in a fatal outcome from contracting the flu. For example, thrombocytopenia can be induced by flu^60^ and flu vaccination^61^. However, we do not know which genes are responsible for thrombocytopenia. Rather, we list 66 candidate genes in Table S2.2 for future investigation.

There were 380 significant comorbidities associated with *Escherichia coli* (taxid 562) (see Table S2.3). 289 involve Certain infectious and parasitic diseases. The top predicted significant comorbid disease for *Escherichia coli* (*E. coli*) was Uveitis. Amazingly, *E.coli* is beneficial for treating Uveitis^62^. Among the 5 interactomes of *E.coli* for Uveitis, deficiency of the top ranked SERPINA1 is a uveitis risk factor^63^. Hypertension, renal tubular acidosis and cardiovascular syphilis were predicted to be other significant comorbidities to *E. coli.* In fact, *E. coli* contamination in drinking water increases the risk of hypertension, renal impairment and cardiovascular disease^64^.

There were 50 significant comorbidities associated with *Salmonella typhimurium* (see Table S2.4). 14 and 10 of them, respectively, are Endocrine, nutritional and metabolic diseases & Diseases of the genitourinary system. The third most significant comorbid disease is psoriasis and Salmonella typhimurium is proposed as photochemotherapy agent for psoriasis^65^. Another example of significant comorbidity is hyperglycemia, which is characterized by high blood sugar. Salmonella infection causes hyperglycemia in pigs^66^.

### On the oncogenic/oncolytic potential of pathogens

The above results (see Tables 1 and 2) show that Neoplasms have the largest relative risk as being caused by viruses and the second largest relative risk as being caused by bacterial infection compared to general causes. Thus, it is worthwhile to examine the oncogenic/oncolytic potential of pathogens in detail.

We define an onco_index, a *p* value characterizing the overlap of pathogen’s interactome with the 723 cancer drivers given in the COSMIC database^67^ to detect pathogens associated with cancers (either oncogenic or oncolytic) from others. An onco_index < 0.05 is considered to have oncogenic/oncolytic potential. Table 5 shows the top 10 cancer associated viruses and bacteria. A complete list of predicted oncogenic/oncolytic pathogens (109 viruses and 15 bacteria) whose onco-index *p* value < 0.05 is given in Supplementary Tables S3 and S4 along with possible MOA proteins, denoted as onco_MOA protein. These are defined as those pathogen proteins that interact with the 723 cancer drivers that have documented literature evidence of oncogenic/oncolytic potential. Of the 124 pathogens 93(75%) have literature evidence, 55(38%) of having oncogenic(oncolytic) potential.

**Table 5:** Top 10 most oncogenic/oncolytic viruses and bacteria ranked by their index.

TaxID	Pathogen name	Onco_index
**Viruses**		
41856	Hepatitis C virus genotype 1	7.53E−10
11706	Human immunodeficiency virus type 1 group M subtype B (isolate HXB2)	1.64E−09
28282	Human adenovirus A serotype 12	3.43E−09
333760	Human papillomavirus type 16	4.64E−09
33708	Murid herpesvirus 4	2.59E−08
28285	Human adenovirus C serotype 5	4.28E−08
11927	Human T-cell leukemia virus 1 (isolate Caribbea HS-35 subtype A)	1.67E−07
11926	Human T-cell leukemia virus 1 (strain Japan ATK-1 subtype A)	4.39E−07
868565	Human herpesvirus 8 type P (isolate GK18)	8.46E−07
10583	Human papillomavirus type 1	2.83E−06
**Bacteria**		
632	Yersinia pestis	4.95E−20
1392	Bacillus anthracis	2.86E−16
177416	Francisella tularensis subsp. tularensis (strain SCHU S4/Schu 4)	1.45E−10
562	Escherichia coli	8.17E−06
623	Shigella flexneri	6.02E−05
1505	Paeniclostridium sordellii	0.000245613
1639	Listeria monocytogenes	0.000552402
272635	Mycoplasma pulmonis (strain UAB CTIP)	0.00150676
622	Shigella dysenteriae	0.002832269
287	Pseudomonas aeruginosa	0.003743935

For those predictions with literature evidence, we have the putative molecular basis of associations that can be validated by experimentalists. About ~ 1/4 of the predictions are novel and worthy of further investigation. To demonstrate how the MOA proteins explain the cancers a given pathogen is associated with, we present the example of the first discovered human tumor virus associated with Epstein-Barr virus^68^. There are 885 interactome proteins for EBV(strain AG876), 64 proteins are cancer drivers according to COSMIC^67^. Among these are EGFR and ERBB2, which are well-known tumor drivers^69^.

### Distinguishing oncolytic from oncogenic pathogens

While the above onco_index distinguishes pathogens associated with cancers from others, some of the cancer associated pathogens can be oncolytic. To distinguish oncolytic from oncogenic potential of pathogens, we examined the interactomes of the 93 pathogens having literature evidence of being oncogenic (total 55) or oncolytic (total 38) and derived oncolytic and oncogenic propensities for each pathogen. First, the oncolytic/oncogenic propensity of an interactome protein of pathogen is derived by calculating a *p* value of its association with oncolytic/oncogenic pathogens. Then the oncolytic/oncogenic propensity of pathogen is calculated as the sum of its interactomes’ propensity (see “Methods” for addition details). A jackknife test was performed on these 93 pathogens by excluding self in deriving oncolytic and oncogenic propensity. We classify a pathogen as being oncolytic if its oncolytic propensity is greater than its oncogenic propensity. The resulting Matthews correlation coefficient (MCC) of this analysis on the 93 pathogens is 0.77, the recall rate (sensitivity) is 94.7%, accuracy 88.2%, and precision is 80.0%. The oncolytic/oncogenic propensity of possible cancer associated pathogens is found in Tables S3 and S4. We then apply the oncolytic/oncogenic propensity to all the pathogens (not limited to those that are predicted cancer associated) to discover possible pathogens that might be used for cancer treatment. The oncolytic_MOA proteins are those human proteins in the given pathogen-human interactome ranked by their oncolytic propensities (see Eq. 3a). 136 pathogens with oncolytic > oncogenic propensity not included in Tables S3 and S4 are listed in Table S5.1 ranked by oncolytic minus oncogenic propensity. Among the top, many are various strains of flu; we note that subtype H5N1 has already shown to have a curative effect on cancer^18^.

In a recent work^26^ we predicted that SARS-CoV-2 is likely associated with cancers by applying the 332 interactomes from ref^70^. Using the same interactomes and the above method, we now additionally predict that SARS-CoV-2 is likely to be oncolytic with a propensity score of 0.017 that will rank 13th in Table S5.1’s 136 predictions. Its significant oncolytic MOA proteins (propensity *p* value < 0.05, see Eq. 3a) along with literature supports are given in Table S5.2. For 10 of the 12 proteins, we have literature evidence of their associations with cancers. For example, MIN, NUP214, PABPC1, LARP4B & DDX10 are established cancer drivers in the COSMIC database^67^. The top protein, MIN, is associated with the risk of colorectal cancer^71^. Knockdown of the second protein MOV10 leads to upregulation of INK4, a tumor suppressor^72^. Inhibition of the third protein NUP214 leads to cell death^73^. PABPC4 plays role in the pathogenesis of colorectal cancer^74^.

While the oncolytic effect might be due to collective effect of the oncolytic proteins, many (48/147) of the top unique pathways (different from those of the oncogenic significant proteins with *p* value < 0.05) of the significant proteins involve PIK3R1 (see Table S6 for unique pathways and proteins involved): e.g., CD28 dependent PI3K/Akt signaling, Signaling by cytosolic FGFR1 fusion mutants, Signaling by PDGFR in disease, etc. PIK3R1 is a known tumor suppressor^75^. Another frequent protein in unique pathways is PIK3CA and its mutations cause a variety of common human tumor types^76^. The above SARS-CoV-2 interactome MOV10 is involved in 12 pathways. Although it does not directly involve apoptotic pathways, its interacting partners ACIN1 and SLC25A5^58^ involve Apoptosis^59^. The colorectal cancer related protein PABPC4 of the SARS-CoV-2 interactome interacts with YWHAQ, YWHAZ & TNFRSF10D that all involve Apoptosis^59^.

### Heart disease and flu

Studies show that heart disease is one of the most common chronic conditions of adults hospitalized with flu^77,78^; it also increases the incidence of strokes. To understand the molecular bases of this observation, we examined the significant comorbidities (q-value < 0.05) belonging to the class “Diseases of the circulatory system” associated with various strains of flu. In total, we predicted 79 pairs of flu virus—Diseases of the circulatory system involving 20 strains of flu and 25 cardiovascular diseases. The 79 pairs and related putative MOA proteins are listed in Table S7.

The 25 comorbid diseases ranked by the number of associated flu strains along with literature evidence are given in Table S8. The top 5 diseases are: *intracranial vasospasm, Dressler's syndrome, brain stem infarction, brain ischemia*^79^*, lymphatic system disease*. We found 12 of the diseases have supporting literature evidence for their associations with flu. The novel predictions of disease associations are useful in guiding clinicians for disease diagnosis.

Next, we analyze the most frequent MOA proteins and their pathways^59^. For each human protein that interacts with flu, we count its frequency as a MOA protein in the 79 flu-heart disease pairs, each of which may contain multiple human proteins that interact with the given strain of flu. The top 100 ranked most frequent proteins are listed in Table S9 along with literature evidence of their association with heart disease. For the top 20 proteins, we find evidence for 9 proteins. For example, the top 1st protein, PIK3R1, is a cardiac regulator^80^. The 2nd protein, GSN, is critical for heart disease^81^.

The 39 significant pathways (q-value < 0.05) involving the above top 100 proteins are given in Table S10. For 22 we found literature evidence of their associations with heart disease. Many of them involve protein synthesis. The top 5 pathways are *Translation*^82^*, Mitochondrial translation initiation*^83^*, Mitochondrial translation elongation*^83^*, Mitochondrial translation termination*^83^*, Mitochondrial translation*^83^. There are number of ribosomal proteins (RPS19/RPL8/RPL30A/RPL3/RPL23/RPL19/RPL15/RPL11) in these pathways that the flu viruses interact with. Studies have shown that mutations in many ribosomal proteins result in a Minute phenotype in *Drosophila* and Cardiomyopathy is correlated with the Minute phenotype^84^. With all the literature evidence, our novel predictions of MOA proteins and pathways are useful in guiding experimentalists for further investigations.

### PHEVIRdb web application

**Figure 1:**
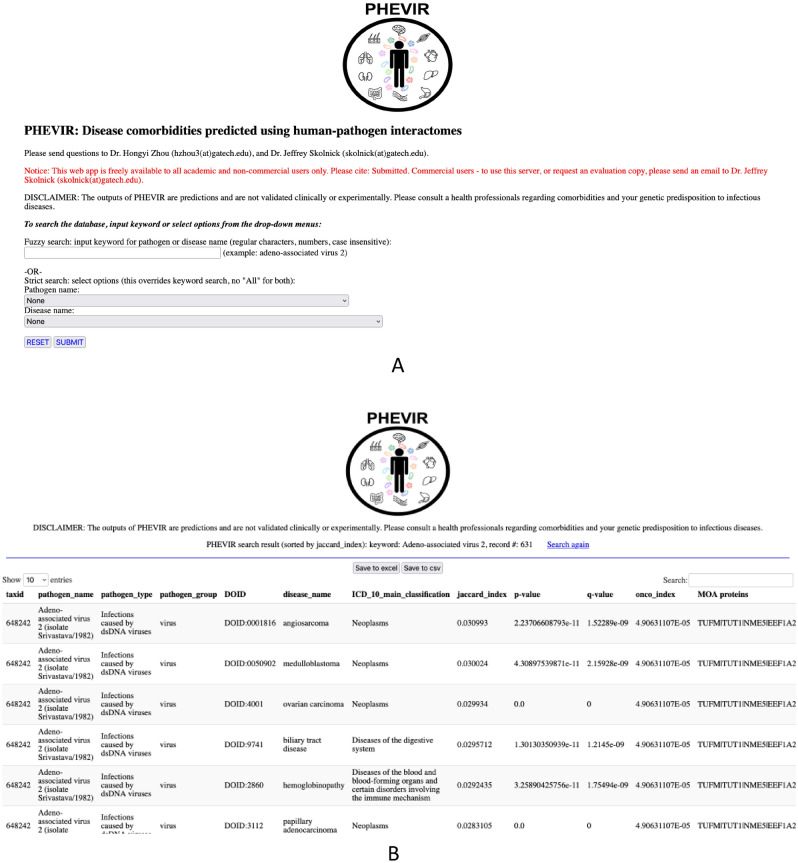
Screenshots of PHEVIR webserver. (**A**) Web interface of PHEVIR. (**B**) Sample output of PHEVIR.

The PHEVIRdb web application allows researchers to access disease comorbidities and the corresponding MOA proteins associated with interactions with the respective pathogen. With multiple input options (keyword and exact search), one can input a keyword for a pathogen name or disease name or select a pathogen name and disease name from the pull down menu. The web service is freely available for academic users at https://sites.gatech.edu/cssb/phevir/. Figure 1 shows screenshots of the web interface and an output example. The keyword search provides a fuzzy search that matches pathogen name or disease name containing the keyword. From the pull down menu, the user can select the pathogen name and disease name for an exact match search. The output can be saved and is searchable by keywords in the search box.

## Discussion

PHEVIR, with 77.2% recall and 37.1% precision based on large scale benchmarking, has predicted post-infection complications of 369 pathogens. Consistent with its quite general definition, our prediction that *Certain infectious and parasitic diseases* have the largest overall relative risk for pathogens. We also predict that *Neoplasms* are the only other group of diseases, on average, having a significant relative risk compared to general causes. By examining some common complex diseases associated with pathogens, oncogenic/oncolytic pathogens and heart disease association with flu, we found that many of PHEVIR’s predictions have literature evidence (which is unknown to the algorithm which views these as *bona fide* predictions). For all predictions, PHEVIR provides the molecular basis of each human disease-pathogen association. In addition, the onco_index and oncolytic propensity can tell whether a pathogen is potentially oncogenic or oncolytic. Importantly, the oncolytic/oncogenic propensity can distinguish oncolytic from oncogenic viruses at 88.2% accuracy and 80.0% precision. Oncolytic pathogens are a useful means of treating cancers and their MOA proteins could be targeted by small molecules or antibodies. Furthermore, PHEVIR predicted 25 heart diseases (Disease of circulatory system) associated with flu for which ~ 50% have literature evidence. These predictions as well as their corresponding MOA proteins are useful for guiding further experimental investigations on disease etiology and for clinic diagnosis. The goal is to eventually find better prevention and treatments of these diseases.

On another note, PHEVIR strongly suggests that many non-Mendelian diseases have a viral component. Indeed, 91% (3285) of the 3608 diseases we consider which cover almost all disease types have a least one viral pathogen associated with it. For the 57 bacteria, 66.7% (2405) of diseases have bacterial associated human disease comorbidities. What is important to realize is that we merely considered 312 strains of viruses and 57 different bacteria. This is clearly a tiny minority of bacterial and viral pathogens. At present, we cannot definitively differentiate whether the pathogen’s infection induces the onset of the complex disease or merely exacerbates its progression. But what this study strongly suggests is that infectious diseases and complex noninfectious diseases are not disjoint and non-interacting. Rather, their interaction is likely to be the rule rather than the exception. Some infections such as an oncolytic virus might be antagonists to the given disease while others, (e.g. oncogenic viruses) might be agonists. This does suggest that one possible means of eliminating/preventing severe diseases such as IBD, AD, and some cancers might be by eliminating the infectious diseases that might be a major contributory factor. In the case of viruses, this suggests that the broader development of antiviral vaccines or antiviral drugs is clearly needed.

## Materials and methods

**Figure 2:**
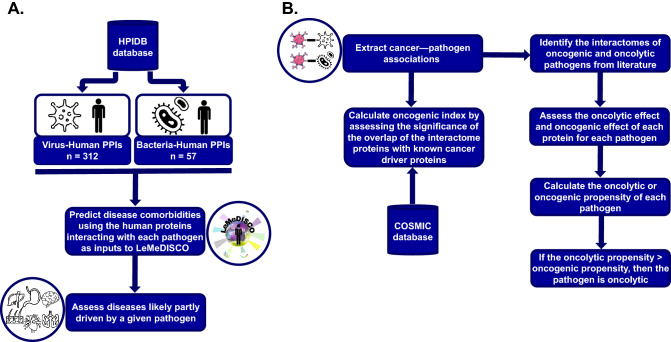
General PHEVIR methodology. (**A**) Comorbidity pipeline; (**B**) Onco-index calculation and oncogenic/oncolytic prediction pipeline.

A flowchart of PHEVIR method is given in Fig. 2. We detail each of the steps below.

### Curating human–pathogen interactomes

Host–pathogen interactome data were extracted from the HPIDB 3.0 database (https://hpidb.igbb.msstate.edu)^22^. Interactions with *homo sapiens* (taxid: 9606) and *homo sapiens* proteins with known UniProtKB IDs were obtained. Next, the pathogens were mapped to their corresponding taxonomy IDs (taxids) using the NCBI taxid file from: https://www.uniprot.org/taxonomy/# which contained 2,658,466 organism entries. Bacteria and viruses were extracted, and those with “No lineage” were removed. The bacteria and viruses were mapped to their corresponding KEGG infectious disease classification from https://www.genome.jp/brite/br08401. If there were less than two *homo sapiens* proteins that interacted with a given pathogen, they were removed as a minimum of 2 proteins are required for the subsequent analysis. There were 312 viruses and 57 bacteria that remaining after filtration.

### Comorbidity predictions

Comorbidities were predicted by LeMeDISCO; we refer the reader to Ref.^21^ for details. Following determination of the significant comorbidities associated with the 312 viruses and 57 bacteria curated from HPIDB, a virus-disease network and a bacteria-disease network were constructed. Following determination of the significant comorbidities associated with the 312 viruses and 57 bacteria curated from HPIDB, a virus-disease network and a bacteria-disease network were constructed.

### Pan-virus and pan-bacteria assessment

We first assessed the frequency of each ICD-10 disease group across all the significant (q-value < 0.05) comorbidities. Then, to find out which ICD-10 disease groups are affected most by pathogens, we define a relative risk (RR) of disease group after infection with respect to background (all possible source of causes):

1 $${\text{RR}} = \frac{{{\raise0.7ex\hbox{${Number\;of\;comorbidities\;by\;group}$} \!\mathord{\left/ {\vphantom {{Number\;of\;comorbidities\;by\;group} {total\;number\;of\;comorbidities}}}\right.\kern-\nulldelimiterspace} \!\lower0.7ex\hbox{${total\;number\;of\;comorbidities}$}}}}{{{\raise0.7ex\hbox{${Number\;of\;diseases\;in\;the\;group}$} \!\mathord{\left/ {\vphantom {{Number\;of\;diseases\;in\;the\;group} {number\;of\;diseases\;in\;the\;library\left( { = 3608} \right) }}}\right.\kern-\nulldelimiterspace} \!\lower0.7ex\hbox{${number\;of\;diseases\;in\;the\;library\left( { = 3608} \right) }$}}}}$$

and calculate a corresponding *p* value using Fisher’s exact test^85^.

### Oncogenic/oncolytic index

For each pathogen an oncogenic/oncolytic index (onco_index) characterized by its *p* value was computed. The *p* value is calculated by Fisher’s exact test^85^ of the overlapped cancer drivers of the COSMIC 723 census proteins^67^ with the interactome proteins. The *p* value is calculated on the following contingency table:

2 $$\left( {\begin{array}{*{20}c} {N^{overlapped} } & {N^{interatome} - N^{overlapped} } \\ {N^{driver} } & {N^{total} - N^{driver} } \\ \end{array} } \right)$$

Here ${N}^{overlapped}$ is the number of overlapped proteins between ${N}^{interatome}$ of interactomes of the given pathogen and the ${N}^{driver}$ = 723 cancer drivers, ${N}^{total}=\mathrm{18,663}$ is the total number of human proteins considered in the work. Onco_MOA proteins are defined as those of the pathogen’s interactomes overlapped with the 723 drivers.

### Oncogenic/oncolytic distinguishing and propensity

To distinguish an oncolytic from oncogenic pathogen, we examine the possible difference between the interactomes (the human partner proteins a pathogen interacts with) of the oncogenic or oncolytic pathogens as evident from literature. We first collect all the interactomes of oncogenic or oncolytic pathogens and then count the frequencies that each human protein is part of the given pathogen-human interactome. Then, using the frequencies of these two lists, we calculate a *p* value^85^ of each protein T’s relative risk for oncolytic or oncogenic effects using the following contingency tables:

3a $$\left( {\begin{array}{*{20}c} {N_{lytic}^{T} } & {N_{lytic}^{all} - N_{lytic}^{T} } \\ {N_{genic}^{T} } & {N_{genic}^{all} - N_{genic}^{T} } \\ \end{array} } \right)$$

3b $$\left( {\begin{array}{*{20}c} {N_{genic}^{T} } & {N_{genic}^{all} - N_{genic}^{T} } \\ {N_{lytic}^{T} } & {N_{lytic}^{all} - N_{lytic}^{T} } \\ \end{array} } \right)$$

where (3a) for oncolytic effect, (3b) for oncogenic effect; ${N}_{lytic}^{T}$,${N}_{genic}^{T}$ are numbers of oncolytic, oncogenic pathogens targeting the protein T; ${N}_{lytic}^{all}$, ${N}_{genic}^{all}$ are total number of oncolytic, oncogenic pathogens. We then define the oncolytic or oncogenic propensity of a pathogen as

4 $${\text{propensity}} = \mathop \sum \limits_{interactomes} - {\text{log}}\left( {p - value} \right)/N_{onco}^{all}$$

The oncolytic propensity is obtained using the *p* value from (3a) and ${N}_{onco}^{all}$ is the total number of unique interactomes of oncolytic pathogens; and the oncogenic propensity from (3b) and ${N}_{onco}^{all}$ is the total number of unique interactomes of oncogenic pathogens. When a pathogen’s oncolytic propensity > oncogenic propensity, we predict it to be an oncolytic pathogen; otherwise it is classified as an oncogenic pathogen. Oncolytic_MOA proteins are defined as the overlap of interactomes of given pathogen with the union of those from literature supported oncolytic pathogens.

## Supplementary Information

Supplementary Information 1.

Supplementary Information 2.

## Data Availability

The datasets generated and/or analysed during the current study are not publicly available due to the tools generating them are independently licensed, but are available from the corresponding author on reasonable request.
