# Supplementary material for: PHEVIR: an artificial intelligence algorithm that predicts the molecular role of pathogens in complex human diseases
Source: Sci Rep. 2022 Dec 3;12:20889. doi: 10.1038/s41598-022-25412-x (PMC9719543; doi:10.1038/s41598-022-25412-x)
Supplement: Supplementary file 1 — Supplementary Information 1. [file 41598_2022_25412_MOESM1_ESM.docx]

**Supplementary Material for**

PHEVIR: An artificial intelligence algorithm that predicts the molecular role of pathogens in complex human diseases

Hongyi Zhou, Courtney Astore and Jeffrey Skolnick

Center for the Study of Systems Biology, School of Biological Sciences, Georgia Institute of Technology, 950 Atlantic Drive, N.W., Atlanta, GA 30332, USA

**Supplementary Tables**

These tables are in Supplementary Excel spreadsheets: Table S1.1, Table S1.2, Table S1.3, Table S1.4, TableS2.1, TableS2.2, TableS2.3, TableS2.4, TableS3, TableS4, TableS5.1, TableS5.2, TableS6, TableS7, TableS8, TableS9, TableS10.
